# Prevalence and determinants of preterm birth at University Teaching Hospital of Kigali: a retrospective study

**DOI:** 10.1186/s12884-026-08739-5

**Published:** 2026-02-07

**Authors:** Israel Cyubahiro Munyambaraga, Césarie Nikuze, Edmond Nsengimana, Placide Shema Niyonshuti, Philemon Manishimwe, Richard Kalisa, Erigene Rutayisire, Pasteur Dushimimana

**Affiliations:** 1 College of Medicine and Health Sciences, School of Public Health, University of Rwanda, Kigali, Rwanda; 2 University Teaching Hospital of Kigali (CHUK), Kigali, Rwanda

**Keywords:** Preterm birth, Determinants, Retrospective study, Maternal health, Neonatal outcomes, University Teaching Hospital of Kigali, Rwanda

## Abstract

**Background:**

Preterm birth, defined as delivery before 37 completed weeks of gestation, is a leading cause of neonatal illness and death worldwide, particularly in low- and middle-income countries such as Rwanda. Despite progress in maternal and newborn healthcare, preterm birth remains a major public health concern. This study aimed to determine the prevalence and determinants of preterm birth.

**Methods:**

This retrospective cross-sectional study reviewed medical records of 1,327 women who delivered at the University Teaching Hospital of Kigali between January 1 and December 31, 2024. Using total enumeration, data were collected on maternal socio-demographic characteristics, obstetric factors, and medical conditions. Descriptive statistics, bivariate analysis, and multivariable logistic regression were applied to determine the prevalence of preterm birth and its associated factors.

**Results:**

The prevalence of preterm birth at the University Teaching Hospital of Kigali was 13.26%. Independent predictors of preterm birth included a history of preterm birth (AOR = 7.13, 95% CI: 3.08–16.49), preeclampsia (AOR = 3.02, 95% CI: 1.32–6.89), hypertension (AOR = 2.13, 95% CI: 1.04–4.35), premature rupture of membranes (AOR = 7.57, 95% CI: 4.06–14.09), and attending four or more antenatal care visits (AOR = 4.19, 95% CI: 1.84–9.56).

**Conclusion:**

Preterm birth remains a major public health concern in Rwanda, particularly among high-risk pregnancies referred to tertiary care centers such as the University Teaching Hospital of Kigali. These findings highlight the need for strengthening early risk detection, improving primary healthcare services, and ensuring timely referral and management of at-risk women to reduce preterm birth and its associated complications.

## Background

Preterm birth (PTB), defined as delivery before 37 completed weeks of gestation, remains a major global public health challenge and the leading cause of neonatal morbidity and mortality worldwide [1–3]. Depending on gestational age, preterm births are classified as moderate to late preterm (32–37 weeks), very preterm (28–32 weeks), and extremely preterm (< 28 weeks), with earlier births carrying higher risks of severe complications and death [2, 4, 5]. In 2020, an estimated 13.4 million babies were born prematurely, contributing to nearly 900,000 deaths among children under five [6, 7]. Reducing this burden is central to achieving Sustainable Development Goal 3, which aims to lower neonatal mortality to 12 per 1,000 live births by 2030 [8].

The burden of PTB is disproportionately high in low- and middle-income countries, particularly in Sub-Saharan Africa and South Asia, which together account for over 60% of global preterm births and 80% of preterm-related deaths [9–11].Across these regions, prevalence estimates of preterm birth vary between 12.3% and 19.5% in Ethiopia, Kenya, Ghana, and Tanzania [2, 4, 12–14]. In the Democratic Republic of Congo, more than 341,000 infants are born preterm annually, with mortality approaching 50% [15].

PTB is multifactorial, resulting from complex interactions between medical, obstetric, biological, behavioral, and socioeconomic determinants [16–21]. Beyond commonly recognized risk factors such as hypertension, diabetes, HIV, anemia, infections, inadequate antenatal care, and a history of miscarriage or abortion, emerging evidence highlights additional etiologies that significantly contribute to preterm birth. Placenta accreta spectrum (PAS), a serious obstetric condition characterized by abnormal invasion of placental tissue into the uterine wall, has been increasingly identified as a cause of medically indicated preterm delivery due to risks of hemorrhage and maternal instability [22]. Similarly, twin and other multifetal pregnancies substantially elevate the risk of both spontaneous and iatrogenic preterm birth as a result of uterine overdistension, shared placental stress, and complications specific to twin gestations [23].

In Rwanda, preterm birth remains a significant public health issue. National estimates suggest between 35,000 and 43,000 preterm births annually, resulting in 2,070 to 2,600 child deaths from related complications [24]. The national prevalence is approximately 13.8%, with rural district hospitals reporting even higher rates of up to 17.5% [16, 24]. Although neonatal mortality declined slightly from 20 per 1,000 live births in 2015 to 19 per 1,000 in 2020, infant mortality has stagnated, highlighting persistent gaps in prevention and care [25].

Previous research in Rwanda has primarily examined preterm birth among high-risk subgroups, such as women with severe preeclampsia or pregnancy-induced hypertension. While these studies provide valuable clinical insights, they focus on narrow populations and cannot be generalized to all preterm births. Evidence on the overall prevalence and broader determinants of preterm birth within tertiary-level settings remains limited.

The University Teaching Hospital of Kigali (CHUK), the largest national referral facility managing high-risk pregnancies and complex obstetric conditions, provides a unique context for understanding patterns of preterm birth in Rwanda. This study aimed to determine the prevalence of preterm birth and to identify socio-demographic, lifestyle, obstetric, and medical factors associated with preterm birth among women who delivered at this institution in 2024. The findings will contribute to evidence-based interventions and support Rwanda’s efforts to accelerate progress toward SDG 3.

## Methods

### Study design

This retrospective cross-sectional study assessed the prevalence and determinants of preterm birth at the CHUK.

### Study setting

This study was conducted at the CHUK, the largest tertiary and referral hospital in Rwanda. The hospital receives maternal referrals from 29 district hospitals across the country and manages a substantial volume of complex and high-risk pregnancies requiring specialized obstetric and neonatal care [26, 27]. The maternity department has approximately 60 beds and performs an estimated 2,000 deliveries annually [26]. The hospital provides comprehensive maternal, newborn, and child health services, including antenatal care, intrapartum management, emergency obstetric interventions, postpartum care, and neonatal intensive care. These services are delivered by a multidisciplinary workforce comprising obstetricians, midwives, nurses, anesthetists, and neonatologists. Its established clinical record system and high case load make it a suitable setting for examining the prevalence and determinants of preterm birth [26, 27].

### Study unit

The study unit was each mother–newborn pair corresponding to a delivery that occurred at the University Teaching Hospital of Kigali between 1 January and 31 December 2024.

### Study population

The study population consisted of all women who delivered at the University Teaching Hospital of Kigali during the study period and had completed secondary data on maternal characteristics, gestational age, and neonatal outcomes.

### Inclusion criteria

Women were eligible for inclusion if they had a singleton pregnancy and delivered at CHUK between 1 January and 31 December 2024, with complete information on maternal characteristics, gestational age, obstetric history, and neonatal outcomes.

### Exclusion criteria

The study excluded twin or other multifetal pregnancies, major fetal congenital anomalies, and any deliveries occurring outside CHUK. Records were also excluded if gestational age was missing or unverifiable, or if early neonatal deaths lacked adequate documentation necessary for.

### Sample size and sampling technique

**Fig. 1:**
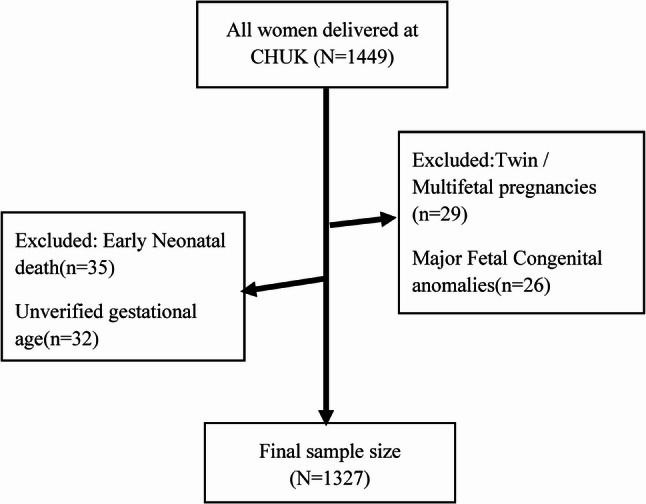
Flowchart of participant selection

The study adopted a total enumeration approach, including all women who delivered at CHUK between 1 January and 31 December 2024 (*N* = 1,449). Following eligibility assessment, multiple exclusions were applied: early neonatal deaths (*n* = 35), unverified gestational age (*n* = 32), twin or multifetal pregnancies (*n* = 29), and major fetal congenital anomalies (*n* = 26). This resulted in a final sample size of 1,327 deliveries (Fig. 1). As total enumeration was used, no sampling technique was required. All eligible medical records were extracted from the hospital database, minimizing selection bias and ensuring a comprehensive representation of maternal and neonatal characteristics for the assessment of preterm birth prevalence and its determinants.

### Data collection procedure and tools

Data were extracted using a structured extraction form designed based on the study objectives to ensure consistency and completeness, and managed through KoboToolbox. To minimize missing data, information was obtained from both patient registries and electronic medical records. A team of trained nurses and midwives conducted the data extraction under the supervision of the principal investigator, ensuring accuracy and adherence to the study protocol.

### Study variables

#### Dependent variable

The dependent variable in this study is preterm birth, defined as delivery before 37 completed weeks of gestation, categorized as “Yes” for births at < 37 weeks and “No” for births at ≥ 37 weeks [28]. Gestational age was primarily determined using the last menstrual period (LMP) as recorded in the maternal file.

#### Independent variables

The independent variables in this study were grouped into socio-demographic, lifestyle, obstetric, and systemic illness factors. Socio-demographic and lifestyle factors included maternal age (< 20, 21–30, 31–40, > 41), residence (urban or rural), marital status (married or unmarried), education level (informal, primary, secondary, or higher), and occupation (employed or unemployed). Obstetric factors considered were gravida (0–2, 3–5, or 6 or more), mode of delivery (cesarean delivery or vaginal birth), history of preterm birth, the use of tocolytics during pregnancy, premature rupture of membranes (PROM), history of miscarriage and preeclampsia. Systematic illness factors included hypertension, diabetes mellitus and infections such as STIs, HIV, or UTIs.

### Data analysis procedures

Data collected using KoboToolbox were analyzed in STATA version 17 following a three-step approach. First, descriptive statistics were used to summarize socio-demographic, lifestyle, obstetric, and systemic illness characteristics using frequencies and percentages. Second, bivariate analysis using chi-square tests was conducted to examine associations between independent variables and preterm birth.

Multivariable modeling strategy: Variables with *p* < 0.20 in bivariate analysis or considered clinically relevant were included as candidate variables in the multivariable logistic regression model. Backward stepwise elimination was applied, retaining variables with *p* < 0.05 [29]. Multicollinearity was assessed using the Variance Inflation Factor (VIF), with VIF < 10 considered acceptable; all variables met this criterion.

This modeling strategy allowed us to account for both statistical significance and clinical relevance in predictor selection, identify independent determinants of preterm birth, and adjust for potential confounders. In the Results section, selected instances where crude odds ratios (COR) and adjusted odds ratios (AOR) differed meaningfully are highlighted to illustrate confounding effects and avoid over-interpretation of attenuated or reversed associations.

### Ethical considerations

This study utilized a data extraction form to collect information from medical records. Ethical approval was obtained from the University of Rwanda Institutional Review Board, operating under the RNEC mandate, with referral number “Ref: CMHS/IRB/399/2025.” Permission to access medical records was also obtained from the University Teaching Hospital of Kigali (CHUK). As the study used secondary data, it posed minimal risk to participants, and all data collected were kept strictly confidential and no personal identifying information was included on the extraction form.

## Results

### Socio-demographic determinant of preterm birth

Socio-demographic characteristics showed notable differences in the distribution of preterm birth. Preterm birth was more frequent among mothers younger than 21 years (30.8%) and those older than 40 years (22.1%) compared with women aged 21–30 years (11.5%) and 31–40 years (13.1%). Women with informal education experienced a higher proportion of preterm birth (19.7%) compared with those with primary (12.7%), secondary (12.0%), or higher education (13.0%). Unemployed women also had a slightly higher proportion (14.2%) compared with employed women (12.1%). Other socio-demographic variables such as residence, marital status, and infant sex showed minimal differences (Table 1).

**Table 1:** Socio-demographic determinant of preterm birth (*N* = 1327)

Variable(s)	Total	Preterm Birth		chi-square tests
		Yes	No	
	*n*(%)	*n* (%)	*n* (%)	*p*-value
Residence				0.158
Rural	414(31.2)	63(15.2)	351(84.8)	
Urban	913(68.8)	113(12.4)	800(87.6)	
Mother’s Age(in year)				0.01**
Mean ± SD ( Min- Max)	32.1 ± 5.5 (14–48)			
< 21	13(1.0)	4(30.8)	9(69.2)	
21–30	524(39.5)	60(11.5)	464(88.5)	
31–40	695(52.4)	91(13.1)	604(86.9)	
> 40	95(7.2)	21(22.1)	74(77.9)	
Marital Status				0.441
Married	1105(83.27)	143(12.9)	962(87.1)	
Unmarried	222(16.73)	33(14.9)	189(85.1)	
Education Level				0.094
Informal education	152(11.5)	30(19.7)	122(80.3)	
Primary	490(36.9)	62(12.7)	428(87.3)	
Secondary	516(38.9)	62(12.0)	454(88.0)	
Higher	169(12.7)	22(13.0)	147(87.0)	
Occupation				0.262
Employed	610(46.0)	74(12.1)	536(87.9)	
Unemployed	717(54.0)	102(14.2)	615(85.8)	

Chi-square test *p*-values: ***p < 0.001, **p < 0.01, *p < 0.05

### Obstetric determinant of preterm birth (*N* = 1327)

Obstetric characteristics demonstrated strong associations with preterm birth. Women with a history of preterm birth had the highest proportion (66.5%), followed by those with premature rupture of membranes (73.9%) and preeclampsia (72.3%). Preterm birth was also more common in women with gravidity ≥ 6 (24.4%) than in those with gravidity 0–2 (13.0%) or 3–5 (11.8%). Cesarean deliveries had a higher proportion of preterm births (17.3%) than vaginal deliveries (10.4%). History of miscarriage (20.2%) and fewer than four ANC visits (39.0%) were also associated with increased preterm birth (Table 2).

**Table 2:** Obstetric determinant of preterm births (*N* = 1327)

Variable(s)	Total	Preterm Birth		chi-square tests
		Yes	No	
	*n* (%)	*n* (%)	*n* (%)	*p*-value
Gravida				0.006**
0–2	700(52.8)	91(13)	609(87)	
(3–5)	541(40.8)	64(11.8)	477(88.2)	
≥ 6	86(6.5)	21(24.4)	65(75.6)	
Mode of Delivery				< 0.001***
Caesarean (c/s)	555(41.8)	96(17.3)	459(82.7)	
Vaginal	772(58.2)	80(10.4)	692(89.6)	
History of Preterm Birth				< 0.001***
No	1121(84.5)	39(3.5)	1082(96.5)	
Yes	206(15.5)	137(66.5)	69(33.5)	
History of Miscarriage				0.004**
No	1154(87.0)	141(12.2)	1013(87.8)	
Yes	173(13.0)	35(20.2)	138(79.8)	
Number of ANC Visits				< 0.001***
< 4	59(4.4)	23(39)	36(61)	
≥ 4	1268(95.6)	153(12.1)	1115(87.9)	
Premature rapture of membrane				< 0.001***
No	1170(88.2)	60(5.1)	1110(94.9)	
Yes	157(11.8)	116(73.9)	41(26.1)	
Tocolytics Used				< 0.001***
No	1155(87.0)	167(14.5)	988(85.5)	
Yes	172(13.0)	9(5.2)	163(94.8)	
Preeclampsia				< 0.001***
No	1150(86.7)	48(4.2)	1102(95.8)	
Yes	177(13.3)	128(72.3)	49(27.7)	

Chi-square test *p*-values: ***p < 0.001, **p < 0.01, *p < 0.05

### Systemic illness determinant of preterm birth

Systemic illnesses also showed significant associations with preterm birth. Women with hypertension had markedly elevated proportions (63.7%), followed by those with maternal infections such as HIV, UTIs, or STIs (71.6%). Diabetes mellitus also demonstrated an increased proportion of preterm birth (21.7%). HIV status alone showed only minimal differences between groups (Table 3).

**Table 3:** Systemic illness determinant of preterm birth(*N* = 1327)

Variable	Total	Preterm Birth		chi-square tests
		Yes	No	
	*n*(%)	*n*(%)	*n*(%)	*p*-value
Chronic Hypertension				< 0.001***
No	1145(86.3)	60(5.2)	1085(94.8)	
Yes	182(13.7)	116(63.7)	66(36.3)	
Chronic Diabetes				0.002**
No	1189(89.6)	146(12.3)	1043(87.7)	
Yes	138(10.4)	30(21.7)	108(78.3)	
Infections				< 0.001***
No	1165(87.8)	60(5.2)	1105(94.8)	
Yes	162(12.2)	116(71.6)	46(28.4)	
HIV/AIDS				0.799
No	1213(91.4)	160(13.2)	1053(86.8)	
Yes	114(8.6)	16(14.0)	98(86.0)	

Chi-square test *p*-values: ***p < 0.001, **p < 0.01, * *p* < 0.05

### Prevalence of preterm birth

**Fig. 2:**
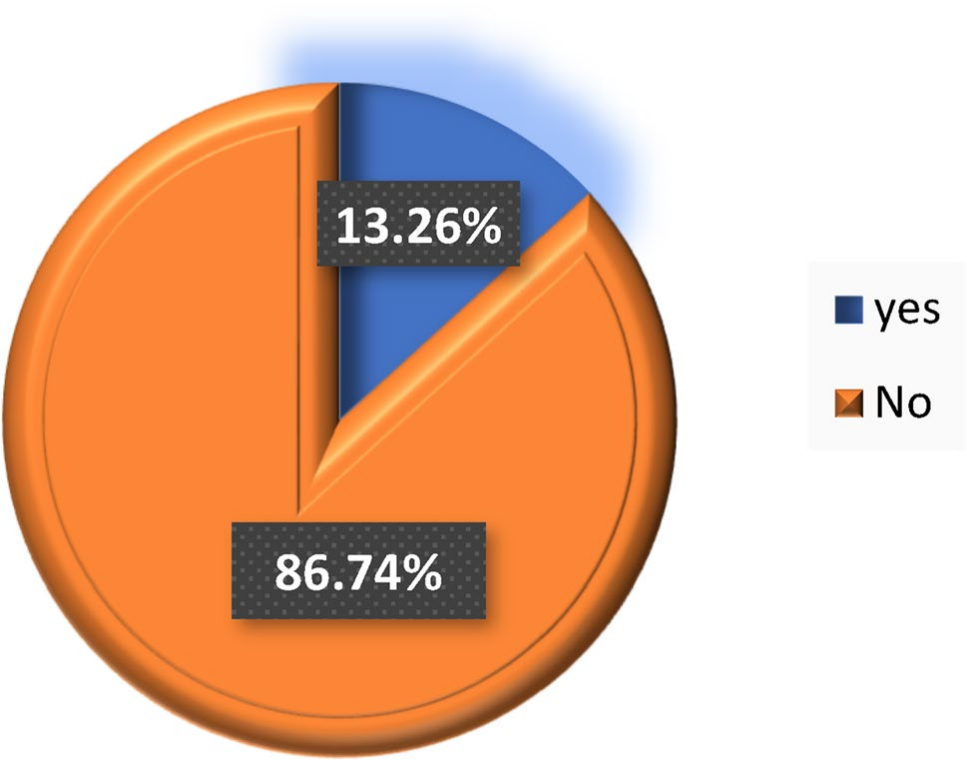
Prevalence of preterm birth

Among the 1,327 total deliveries reviewed, the overall prevalence of preterm birth was 13.3%, while 86.7% were term births (Fig. 2).

### Bivariate and multivariable logistic regression analysis of determinants of preterm birth

Variables with *p* ≤ 0.20 in bivariate analysis were entered into a multivariable logistic regression model using backward elimination, with variables retained at *p* < 0.05 (Table 4). After adjustment, a history of preterm birth (AOR 7.13; 95% CI 3.08–16.49), premature rupture of membranes (AOR 7.57; 95% CI 4.06–14.09), pre-eclampsia (AOR 3.02; 95% CI 1.32–6.89), and chronic hypertension (AOR 2.13; 95% CI 1.04–4.35) remained independently associated with preterm birth.

**Table 4:** Bivariate and multivariable logistic regression analysis of determinants of preterm birth

Variable	Bivariate logistic regression	multivariable logistic regression
	COR[95%CI]	AOR [95%CI]
History of preterm birth		
No	1	1
Yes	55.08[35.79–84.76]***	7.13[3.08–16.49]***
Pre eclampsia		
No	1	1
Yes	59.97[38.69–92.94]***	3.02[1.32–6.89]**
Antenetal care visit		
< 4	1	1
≥ 4	0.21[0.12–0.37]***	4.19[1.84–9.56]***
Chronic Hypertension		
No	1	1
Yes	31.78[21.33–47.34]***	2.13[1.04–4.35]*
Premature rapture of membrane		
No	1	1
Yes	52.34[33.68–81.33]***	7.57[4.06–14.09]***

*COR *Crude Odd Ratio, *AOR* Adjusted Odd Ratio. *P < 0.05 **P < 0.01 ***P < 0.001

Attendance of ≥ 4 antenatal care (ANC) visits was inversely associated with preterm birth in the crude analysis (COR 0.21) but showed a positive association after multivariable adjustment (AOR 4.19). Similarly, pre-eclampsia demonstrated a markedly larger crude association (COR 59.97) compared with the adjusted estimate (AOR 3.02). These differences reflect the presence of confounding in unadjusted analyses, with adjusted odds ratios representing associations after controlling for potential confounders.

## Discussion

This study found a preterm birth prevalence of 13.26% at the University Teaching Hospital of Kigali, a figure that is clinically important and consistent with the burden reported across Sub-Saharan Africa. Although slightly higher than the global average of 10.6% reported by the World Health Organization, this prevalence falls within the regional range of 12–18%, where preterm birth remains a major contributor to neonatal morbidity and mortality [30, 31]. When compared to neighboring countries, where PTB prevalence ranges from 10.4% to 17.9% [32–34], the rate observed at CHUK appears consistent with broader regional trends. Within Rwanda, this prevalence is slightly lower than that reported in a rural district hospital (17.5%) and comparable to findings from a national longitudinal study (13.8%) [16, 24]. The relatively elevated prevalence at CHUK may be explained by its role as a tertiary referral center, which receives a disproportionate number of high-risk pregnancies and complicated obstetric cases from lower-level health facilities.

Beyond prevalence, this study identified a prior history of preterm birth as the strongest independent predictor of recurrence, with affected women being more than six times as likely to deliver preterm again. Similar associations have been consistently documented across Sub-Saharan Africa, particularly in Eastern African countries such as Ethiopia, Kenya, and Tanzania, underscoring the substantial recurrence risk among women with a previous preterm delivery [10, 31, 32, 35]. This recurrent pattern may be attributed to persistent biological and clinical factors, including uterine or cervical structural anomalies, chronic maternal conditions, and sustained inflammatory responses [36, 37].

In addition, preterm rupture of membranes (PROM) was associated with more than a sixfold increase in the risk of preterm birth. This finding is consistent with evidence from Kenya, Ethiopia, and Tanzania, where similar strong associations between PROM and preterm delivery have been reported [31, 34, 38]. The underlying mechanisms are likely multifactorial and may involve ascending infections, chronic intrauterine inflammation, or premature weakening of the fetal membranes related to nutritional deficiencies or untreated infections [39]. Given CHUK’s role as a tertiary referral hospital, it is plausible that many women presenting with PROM had already experienced delayed referrals or suboptimal management at peripheral facilities, further compounding their risk of early delivery [40–42].

An unexpected finding of this study was the association between attending four or more ANC visits and a higher likelihood of preterm birth. This contrasts with studies from Tanzania and Ethiopia, which reported that more frequent ANC attendance was associated with a reduced risk of preterm delivery [34, 38]. This discrepancy likely reflects reverse causation, whereby women with complications or known high-risk pregnancies receive more intensive monitoring and attend ANC more frequently, rather than increased ANC visits causing preterm birth. Additionally, evidence from Rwanda indicates that only high-quality ANC not the number of visits alone is linked to improved birth outcomes [43]. At CHUK, this association is further influenced by its status as a referral hospital, where women often present after multiple ANC visits at lower-level facilities, frequently when complications have already developed. These findings suggest that emphasis should shift from quantity to quality of antenatal care, ensuring timely risk identification and effective management during each visit [20].

Women with hypertension were more than twice as likely to deliver preterm, with an even higher risk among those with preeclampsia, consistent with studies from Tanzania, Ethiopia, and Ghana [34, 38, 44]. At CHUK, hypertensive disorders account for nearly one-third of severe obstetric referrals, often due to delayed detection and management [41, 45]. Part of the observed association may reflect iatrogenic preterm births, where labor is induced or cesarean delivery performed before 37 weeks due to deteriorating maternal or fetal status, particularly in severe preeclampsia, rather than spontaneous preterm labor [45]. As these risks are largely preventable, strengthening antenatal care including routine blood pressure monitoring, timely diagnosis, prompt referral, use of low-dose aspirin for high-risk women, and empowering frontline providers could help reduce preterm birth and improve maternal and neonatal outcomes [46].

In addition to hypertensive disorders, lifestyle-related conditions such as gestational diabetes mellitus also influence pathways leading to preterm birth [47]. Although this study could not fully evaluate GDM due to inconsistent documentation, global evidence shows that structured physical activity, weight management, and dietary improvements, such as reducing refined carbohydrates and increasing fiber intake, significantly reduce the likelihood of gestational diabetes and improve glycemic control during pregnancy [48]. These lifestyle measures may indirectly reduce the risk of preterm birth associated with poorly controlled metabolic conditions. Integrating nutrition and physical activity counseling more deliberately into routine antenatal care may therefore offer an important opportunity to improve maternal risk management in Rwanda.

Although this study did not measure the use of antenatal corticosteroids or magnesium sulfate (MgSO₄), these interventions remain central to the evidence-based management of women at risk of preterm birth. Antenatal corticosteroids are strongly recommended by the World Health Organization to accelerate fetal lung maturation and reduce neonatal complications such as respiratory distress syndrome, intraventricular hemorrhage, necrotizing enterocolitis, and early neonatal mortality among infants born before 34 weeks’ gestation [49]. Similarly, MgSO₄ administered to women at risk of imminent early preterm birth (< 32 weeks) provides significant fetal neuroprotection and reduces the risk of cerebral palsy and severe motor disability². In our study, inconsistent documentation in medical records prevented assessment of the use or timing of these therapies. Strengthening adherence to national and WHO guidelines on antenatal corticosteroids and MgSO₄ could improve perinatal outcomes and enhance preterm birth management in tertiary facilities such as CHUK [50].

Altogether, the results highlight that preterm birth at CHUK is driven by a complex interaction of maternal obstetric history, underlying health conditions, and health service factors. The consistency of these risk factors across studies in Sub-Saharan Africa suggests the need for a harmonized approach to maternal care. Strengthening ANC platforms with a focus on risk assessment, continuity of care, and clinical decision-making capacity is essential to reducing preterm birth across similar settings. The findings also align with Rwanda’s national maternal health strategies, which emphasize early detection of pregnancy complications, improved referral pathways, and equitable access to high-quality ANC services, especially in rural districts.

## Strength and limitation

This study has several limitations to consider when interpreting the findings. First, being conducted at a tertiary referral hospital that primarily manages high-risk and complicated pregnancies, the prevalence and determinants of preterm birth observed here may not fully represent the general population. Second, the retrospective use of medical records introduced the potential for missing, incomplete, or inaccurately documented information, particularly for gestational age, lifestyle and behavioral factors, and interventions such as antenatal corticosteroids and magnesium sulfate. Gestational age was mainly estimated using last menstrual period (LMP), which is subject to recall error and inconsistent recording. Overestimation of gestational age would tend to underestimate the true prevalence of preterm birth by misclassifying some preterm deliveries as term, whereas underestimation would inflate the preterm prevalence by classifying term births as preterm. Similarly, misclassification of gestational age could attenuate observed associations toward the null or, less commonly, exaggerate odds ratios, depending on whether errors occurred differentially across exposure groups. In contrast, studies using early ultrasound dating provide more accurate gestational age assessment and more robust prevalence and risk estimates.

Preterm birth was analyzed as a single category, and stratification into early, moderate, and late preterm births was not possible due to inconsistent documentation, limiting exploration of gestational age–specific risk factors. Important determinants, including maternal nutrition, physical activity, diet, alcohol use, smoking, stress, and socioeconomic conditions, were not routinely captured. Some clinical categories, such as diabetes and hypertension, lacked detailed sub-classification, potentially limiting the precision of risk estimates.

Despite these limitations, this study’s strengths include a large sample from Rwanda’s major referral hospital and the use of rigorous statistical methods, including adjusted logistic regression, which strengthened the reliability of identified associations. These findings provide meaningful evidence to guide maternal health policies and clinical practice in Rwanda and similar settings.

## Study implication and future research

The findings underscore the need to strengthen early identification and management of high-risk pregnancies in Rwanda, particularly among women with a history of preterm birth, hypertensive disorders, or premature rupture of membranes. Improving the quality of antenatal care, ensuring consistent use of evidence-based interventions such as antenatal corticosteroids and magnesium sulfate, and strengthening clinical documentation could help reduce preventable neonatal complications. Integrating lifestyle counseling on diet, weight control, and physical activity into routine ANC may further address metabolic risks and improve maternal–fetal outcomes. These findings suggest that CHUK and similar tertiary hospitals should adopt structured ANC quality-improvement audits and strengthen communication channels with rural and urban referring facilities.

Future research should involve multiple hospitals to improve generalizability and collect prospective data on behavioral, nutritional, and psychosocial factors that were not available in routine medical records. Additional research should incorporate fetal and placental conditions, evaluate ANC quality, and assess adherence to national and WHO guidelines for preterm birth management. Follow-up studies are also needed to evaluate whether implementing strengthened ANC practices, early screening strategies, and guideline-based interventions leads to measurable reductions in preterm birth rates. Studies should also classify preterm births into early, moderate, and late categories to better understand how risk factors vary across gestational age groups and to guide more targeted interventions.

## Conclusion

Preterm birth remains a significant public health challenge in Rwanda. At CHUK, a history of preterm birth, premature rupture of membranes (PROM), and hypertensive disorders were the strongest predictors, highlighting the need for early identification and close monitoring of high-risk pregnancies. The association between four or more antenatal visits and increased preterm birth likely reflects delayed recognition or inadequate management of complications before referral.

To address these challenges, interventions should be implemented across the health system. At the community and health center level, a high-priority action is the enforcement of minimum ANC documentation standards, including accurate recording of gestational age, blood pressure, and PROM status at every visit. At district hospitals, priority should be given to timely referral coupled with the early initiation of antenatal corticosteroids and magnesium sulfate according to national protocols. At tertiary hospitals, systematic and protocol-driven use of antenatal corticosteroids and magnesium sulfate, alongside advanced neonatal care for preterm infants, should be consistently implemented. These actions align with WHO Quality of Care standards and SDG 3 targets and can reduce preventable preterm births while improving maternal and neonatal outcomes in Rwanda [51].

## Data Availability

The datasets generated and/or analyzed during the present study are available from the corresponding author upon reasonable request.

## Abbreviations

ANC: Antenatal Care
AOR: Adjusted Odds
CHUK: University Teaching Hospital of Kigali
CI: Confidence Interval
COR: Crude Odds Ratio
IRB: Institutional Review Board
LMICs: Low- and Middle-Income Countries
PTB: Preterm Birth
PROM: Premature Rupture of Membranes
RNEC: Rwanda National Ethics Committee
SDG: Sustainable Development Goal
SPH: School of Public Health
STIs: Sexually Transmitted Infections
UTI: Urinary Tract Infection
VIF: Variance Inflation Factor
WHO: World Health Organization
